# Status of vitamin D and parameters of calcium homeostasis in renal transplant recipients in Nepal: a cross sectional study

**DOI:** 10.1186/s12882-018-1088-x

**Published:** 2018-10-22

**Authors:** Santosh Timalsina, Mahesh Raj Sigdel, Santosh Baniya, Shantos Subedee

**Affiliations:** 1 Department of Biochemistry, Chitwan Medical College, Bharatpur, Nepal; 2 Department of Nephrology, Tribhuvan University Teaching Hospital, Kathmandu, Nepal; 3 Department of General Practice and Emergency Medicine, Tribhuvan University Teaching Hospital, Kathmandu, Nepal; 4 National Public Health Laboratory, Kathmandu, Nepal

**Keywords:** Hypovitaminosis D, Renal transplant recipients, Sensible sun exposure

## Abstract

**Background:**

Vitamin D**,** apart from being an important part of the “calcium-vitamin D-parathyroid hormone” endocrine axis, has diverse range of “non-calcemic” biological actions. A high prevalence of vitamin D deficiency has been observed in renal transplant recipients (RTRs) worldwide. This study aimed to determine the prevalence of hypovitaminosis D in Nepalese RTRs and interrelations between serum 25-hydroxyvitamin D [25(OH) D] and other biochemical parameters.

**Methods:**

A total of 80 adult RTRs visiting a university hospital were enrolled in this cross sectional study. Serum 25(OH) D and intact parathyroid hormone (iPTH) were measured using Enhanced Chemiluminiscent Immunoassay. The RTR population was categorized into recent transplant recipients (≤1 year) and long term recipients (> 1 year). The vitamin D status was defined as per NKF/KDOQI guidelines. SPSS version 20.0 was used to analyze the data. Appropriate statistical tests were applied to compare variables between groups and establish correlation. *P* < 0.05 was considered to be statistically significant.

**Results:**

The mean age of the recipients was 38.11 ± 11.47 years (68 males, 85.0%). Chronic glomerulonephritis was the leading cause of CKD. The two RTR groups (recent and long term) didn’t differ in demographic and biochemical characteristics. 83.75% of the recipients had PTH levels above the upper limit of the recommended range for their stage of CKD. 57.5% had hypocalcemia and none of the recipients had hypercalcemia. The median serum 25(OH) D was 24.15 ng/ml (8.00–51.50 ng/ml). Only 27.5% had sufficient vitamin D status whereas 53.8% were vitamin D insufficient and 18.8% were vitamin D deficient, the distribution almost comparable in the 2 transplant group. The serum 25(OH) D was not significantly affected by the time post-transplant, gender and sunlight avoidance. There was a significant negative correlation between serum 25(OH) D and iPTH (Pearson’s *r* = − 0.35, *P* = 0.001), but not so with the graft function.

**Conclusion:**

There is a high prevalence of vitamin D insufficiency in RTRs. The deficiency status is not corrected despite of nutritional improvement and normalization of GFR post-transplantation and likely exacerbates secondary hyperparathyroidism. Vitamin D supplementation coupled with sensible sun exposure could be important strategies in optimization of the vitamin D status in this population.

## Background

Vitamin D, a fat soluble vitamin, is an important part of the “calcium-vitamin D-parathyroid hormone” endocrine axis that plays a crucial role in the calcium homeostasis [1]. Inadequate serum vitamin D is associated with secondary hyperparathyroidism, increased bone turnover, and bone loss, which increase fracture risk [2]. Currently, the focus has shifted to its’ non-calcipotropic roles such as induction of cell differentiation, inhibition of cell growth, immunomodulation, and control of other hormonal systems among many others, as supported by the wide distribution of the enzyme 1-α hydroxylase and vitamin D receptors (VDRs) in more than 30 different tissues [3]. A plethora of genetic, nutritional, and epidemiological evidence link vitamin D deficiency with disorders unrelated to calcium homeostasis such as hypertension, disturbed muscle function, susceptibility to infections, autoimmune diseases (Crohn’s disease, multiple sclerosis, rheumatoid arthritis and type I diabetes mellitus) and specific cancers (prostate, colon and breast cancers) [4].

Using serum 25-hydroxyvitamin D concentration [25(OH) D] for defining vitamin D status, a high prevalence of vitamin D deficiency, varying between 50 and 90%, has been found in general population of both developed and developing nations [1, 5]. The deficiency, expectantly common in patients with Chronic Kidney Disease (CKD) [6], is also of common occurrence in renal transplant recipients (RTRs), even months or years after transplantation [7, 8]. The possible explanations for this high prevalence in the transplant group are diverse [9, 10]. Persistent hyperparathyroidism is another issue with RTRs that is likely aggravated by vitamin D deficiency, and it has been observed to persist in a significant majority of the recipients and has potential negative consequences on skeletal health and even on the graft function [11]. Furthermore, the immunomodulatory role of vitamin D makes it even more pertinent to renal transplantation.

The transplant service started in Nepal from 2008, and since then, there have been significant inroads in its provision to Nepalese population in need at selected centers in Nepal. Most of the prior studies have been focusing on the Caucasian population, and our study is one of the very few of its kind in this part of world. Even though hypovitaminosis D is very common, there is a weak evidence and insufficient data to support routine vitamin D supplementation in RTRs as per the recent guidelines. [12].

This study aimed to examine the prevalence of hypovitaminosis D and interrelations between serum levels of 25(OH) D, iPTH and other biochemical parameters so as to provide an overall outlook of the calcium homeostasis in adult Nepalese RTRs.

## Methods

### Study design and study population

After receiving ethical approval from Institutional Review Board, Institute of Medicine [Ref #295(6–11-E)], this cross-sectional observational study was performed between November 2015 – April 2016 and consisted of a total of 81 adult RTRs (> 18 y of age) out of the total 130 RTRs available at the commencement of the study (sampling percentage: 62.3%) following up in the renal transplant outpatient department (OPD) under Nephrology Unit of Department of Internal Medicine, Tribhuvan University Teaching Hospital, Kathmandu (Latitude 27^0^ 42′ 2.7684" N), Nepal. There is seasonal variation in the mean monthly sunshine duration in this city; Pre-monsoon and post-monsoon seasons have higher mean monthly sunshine duration (about 8 h/day) than summer (about 5 h/day) and winter season (about 7 h/day) [13].

Exclusion criteria were patients with acute illness, mental disorders, need for dialysis, prior hyperparathyroidism, receiving any vitamin D compounds (ergocalciferol, cholecalciferol, and alphacalcidiol) after transplantation and patients who received bisphosphonates, corticosteroids prior to transplantation. An interviewer-administered questionnaire was filled out during outpatient visit after the written consent from research participants and their blood samples (5 ml) were drawn under aseptic conditions by trained laboratory personnel.

### Immunosuppressive regimen

All the recipients were under “triple regimen” immunosuppression that included the combination of steroid (prednisolone), tacrolimus and mycophenolate mofetil.

### Biochemical measurements

Non-fasting blood samples were drawn and collected between 10 00 and 14 00. Upon arrival at the laboratory within 2 h, the blood samples were centrifuged at 4000 rpm for 5 min, aliquotted and stored at -20 °C until analysis. Laboratory variables included measurements of serum creatinine, albumin, phosphate, calcium, alkaline phosphatase, 25-hydroxy vitamin D [25(OH) D] and intact parathyroid hormone (iPTH). Serum 25(OH) D and iPTH were measured using Vitros ECi™ analyzer (Ortho Clinical Diagnostics, Rochester, NY) that used an enhanced chemiluminiscence immunoassay technology. Rest of the laboratory parameters were assayed by a semi-automated system (Biotecnica Chemistry Analyzer 3000, Italy) utilizing spectrophotometric technique.

### Estimated glomerular filtration rate (eGFR)

It was calculated using the four variable abbreviated form of the Modification of Diet in Renal Diseases (MDRD) study equation [14] eGFR = [32,788 × Serum creatinine (μmol/L) ^-1.154^ × Age ^− 0.203^ × 0.742 (if subject is female) × 1.212 (if black)] ml/min/1.73 m^2^.

### Staging of CKD

The staging of CKD and target PTH level for the respective stages was done following NKF/KDOQI guidelines [15]. RTRs were categorized as recent transplant recipients (≤ 1 year since transplant) or long-term transplants (> 1 year post-transplant) [16].

### Sun exposure

Patients were categorized into 1 of 3 groups (complete sun avoidance, partial sun avoidance or no sun avoidance) according to a score obtained by asking a set of 3 questions concerning avoidance of sun exposure that has been validated elsewhere [17].

### Serum calcium

Serum calcium (Ca) level (mmol/L) was adjusted according to value of serum albumin according to the equation: Corrected Ca = measured Ca + 0.02[40- albumin level (g/L)]. Hypercalcemia and hypocalcemia were defined as serum Ca > 2.6 mmol/L and < 2.1 mmol/L respectively.

### Vitamin D status

The vitamin D status was defined as per NKF/KDOQI guidelines [15]. It was considered adequate when serum 25(OH) D concentrations was > 30 ng/ml (> 75 nmol/l, Conversion factor for 25(OH) D: 1 ng/ml = 2.5 nmol/l). Concentrations between 16 and 30 ng/ml (40–75 nmol/l) represented vitamin D insufficiency. Vitamin D deficiency was defined as serum 25(OH) D concentration ≤ 15 ng/ml (< 37.5 nmol/l).

### Statistical analysis

Data analysis was performed using SPSS version 20.0 (IBM Corporation, Armonk, NY, USA). Analyses included standard descriptive statistics with normally distributed variables expressed as mean ± standard deviation and non-normally distributed variables as medians (range). Unpaired t-test or Mann-Whitney U test was used for comparison of means or medians as appropriate and chi-square test for comparison of proportions between variables. Relationship between variables was examined using Pearson’s correlation analysis. A *p*-value < 0.05 was considered statistically significant.

## Results

**Fig. 1:**
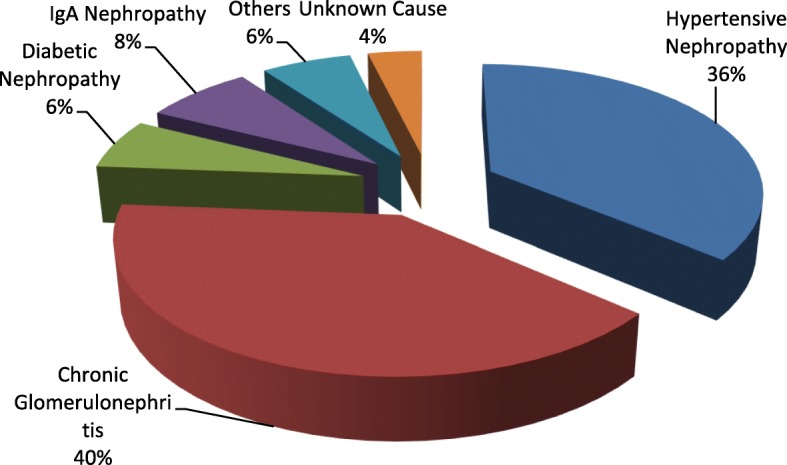
Causes of CKD in the renal transplant population

There were a total of 80 RTRs in our study population included in the analysis out of 81. One of them was excluded because of his need for dialysis. There were higher number of males (*n* = 68; mean ± SD age: 38.54 ± 11.95 years) compared to females (*n* = 12; mean ± SD age: 35.67 ± 8.23 years) in our study population (*n* = 80). Chronic Glomerulonephritis was the leading cause of CKD that warranted renal transplantation (40%) followed by Hypertensive Nephropathy (36%). A minority of the cases (6%) were due to other causes such as Focal Segmental Glomerulosclerosis, Polycystic Kidney Disease, Obstructive uropathy and Lupus nephritis [Fig. 1]. 87.5% of the recipients had partial avoidance to sunlight and 12.5% of them didn’t avoid sunlight at all. There were no subjects who completely avoided sunlight. None of the participants were current smoker or alcohol consumer. The recipient groups (recent vs. long term) didn’t differ significantly in demographic and biochemical characteristics [Tables 1 and 2].

**Table 1:** Demographic and transplantation characteristics of the study population expressed as mean (± SD) or median (range) as possible

Variable	Recently transplanted recipients (*n* = 41)	Long term transplant recipients (*n* = 39)	*P* -value
Age (years)	36.12 ± 10.13	40.21 ± 12.52	NS
Gender (Male/Female)	36/5	32/7	NS
Weight (kg)	58.41 ± 10.55	60.46 ± 9.19	NS
Height (cm)	163.93 ± 6.62	164.1 ± 8.25	NS
BMI (kg/m^2^)	21.72 ± 3.60	22.43 ± 2.90	NS
Time post-transplantation (months)	5.93 ± 2.50	30.51 ± 13.65	< 0.001
Pre-transplant dialysis (months)	5.00 (0.00–24.00)	5.00 (0.00–18.00)	NS
Sunlight avoidance (Partial/No avoidance)	36/5	34/5	NS

**Table 2:** Serum biochemical characteristics of the study population expressed as mean (± SD) or median (range)

Variable	Recently transplanted recipients (*n* = 41)	Long term transplant recipients (*n* = 39)	*P* -value
Corrected calcium (mmol/l)	2.03 ± 0.17	2.00 ± 0.22	NS
Phosphate (mmol/l)	0.97 ± 0.14	0.97 ± 0.20	NS
Alp (U/l)	233.83 ± 72.03	214.72 ± 66.97	NS
Creatinine (μmol/l)	126.39 ± 20.47	125.13 ± 39.53	NS
eGFR (ml/min/1.73 m^2^)	59.73 ± 11.44	61.05 ± 16.39	NS
iPTH (pg/ml)	75.40 (17.50–206.60)	54.30 (26.10–405.80)	NS
25(OH) D (ng/ml)	24.60 (8.00–51.50)	22.30 (8.00–47.90)	NS

The mean corrected serum calcium in the transplant population was 2.02 ± 0.19 mmol/L. 57.5% had hypocalcemia and there were no recipients with hypercalcemia. The mean serum phosphate was 0.97 ± 0.17 mmol/land 18.8% of the transplant population had hypophosphatemia. The median iPTH in the transplant population was 64.25 pg/ml (17.5–405.8 pg/ml). 83.75% of the recipients had PTH levels above the upper limit of the recommended range for their stage of CKD. There was a significant negative correlation between serum calcium and iPTH (Pearson’s *r* = − 0.43, *P* < 0.001) but not so with serum phosphate.

**Fig. 2:**
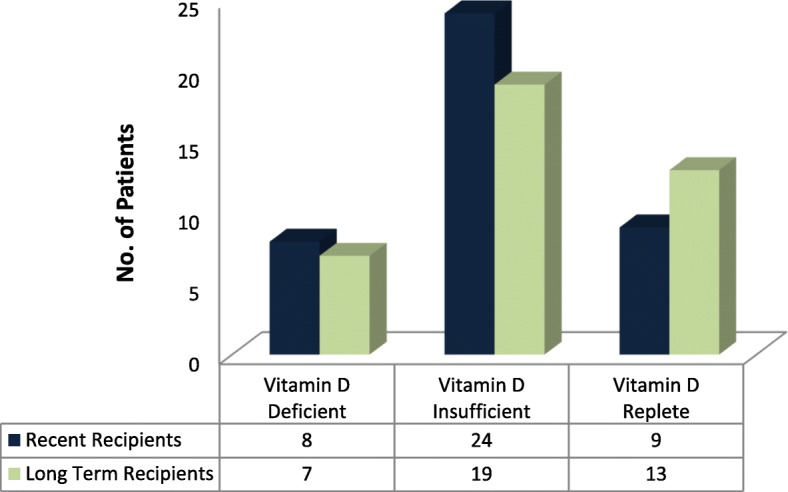
Vitamin D status of the total transplant population based on post-transplant status (expressed as numbers)

The median serum 25(OH) D in the transplant recipients was 24.15 ng/ml (8.00–51.50 ng/ml). Only 27.5% had sufficient vitamin D status whereas 53.8% were vitamin D insufficient and 18.8% were vitamin D deficient, the distribution almost comparable in the 2 transplant groups **[**Fig. 2**].** The serum 25(OH) D was not significantly affected by the transplant status (recent vs. long term transplant), gender and BMI. The partial sun avoidance group had lower median serum 25(OH) D than no avoidance group, but was statistically insignificant (23.65 vs. 24.55 ng/ml, *P* = 0.735).

Table 3 shows the correlation of serum 25(OH) D with other biochemical parameters when all the RTRs were considered. A significant negative correlation (*P* < 0.01) between 25(OH) D and iPTH was observed. Graft function, as indicated by eGFR, didn’t have significant relationship with 25(OH) D.

**Table 3:** Correlation of 25(OH)D with other biochemical parameters in RTRs

Parameters	Correlation coefficient (*P*-value)
iPTH (pg/ml)	− 0.354 (< 0.001)
eGFR (ml/min/1.73 m^2^)	− 0.104 (NS)
Alp	− 0.175 (NS)
Phosphate	− 0.093 (NS)
Calcium	0.174 (NS)

## Discussion

There is a general consensus that the serum 25(OH) D is the best indicator of the vitamin D status of an individual and contributes to majority of the total vitamin D activity because of its higher serum levels than circulating levels of 1,25(OH)_2_D_3_ (almost 1000 times higher) [18]. Furthermore, CKD patients have diminished serum 1,25(OH)_2_D_3_ because of minimal renal 1-alpha hydroxylase activity and this is particularly relevant for RTRs who previously had CKD stage 5 and are CKD patients despite having renal allograft. Recent guidelines have suggested correction of vitamin D insufficiency and deficiency for the CKD patients at different stages using the treatment strategies for the general population. It has also been suggested to consider vitamin D supplementation in stable RTRs with low bone mineral density in the first 12 months, which could further be influenced by the presence of CKD-MBD (Mineral Bone Disorders) in these patients [12].

The prevalence of vitamin D deficiency is very high in the general population of Asia and middle-east countries- the attributed risk factors include: more pigmented skin, the wearing of well-covering clothes, a diet low in vitamin D content and unawareness/unwillingness of supplementation [19]. RTRs are specific risk groups for vitamin D deficiency and it is evidenced by the fact that a significant proportion of our study population had vitamin D deficiency status (53.8% vitamin D insufficient and 18.8% vitamin D deficient), which is comparable to studies elsewhere [16, 20]. The causes that might account for this high prevalence include a) low vitamin D status in the CKD patients (“the past of renal transplant recipients”**)** owing to reduced sun exposure and consequently decreased endogenous synthesis, urinary loss of vitamin D binding proteins and insufficient vitamin D supplementation during dialysis and after transplantation (b) recommended reduced sun exposure because of the association of immunosuppressive therapy in RTRs with skin cancers [9] (c) induction of catabolism of 25(OH) D by immunosuppressive drugs especially glucocorticoids and residual Fibroblast growth factor-23 (FGF-23) activity, that cause increased 24-hydroxylation of 25(OH) D into the inactive metabolite [24,25(OH)_2_D] [10].

Ultraviolet B radiation (UV-B, 280–320 nm) is the only part of the solar UV radiation (290–400 nm) that causes vitamin D synthesis in the skin, and is believed to provide more than 90% of vitamin D required by the body. However, the risk of acquiring skin cancers appears to increase in the RTRs with a history of high sun exposure after transplant, which proportionately increases with the level of immunosuppression [21]. Calcineurin inhibitors e.g. cyclosporine and azathioprine have especially been linked with development of skin cancers in RTRs [22], but the risk with newer immunosuppressants (tacrolimus and mycophenolate mofetil) that are being used in Nepalese RTRs, is still unknown. But again, complete avoidance of sun exposure causes vitamin D deficiency in the RTRs as evidenced by a study that demonstrated significantly lower concentration of serum 25(OH) D in sun avoiders compared to non-avoiders [17]. Our study, however, couldn’t show this association because there were no subjects who completely avoided sunlight (87.5% partial avoidance, 12.5% no avoidance at all with 0% complete avoidance) because it’s not routine in this institute to advise the RTRs to avoid sunlight, and also that sun exposure after morning meal is culturally considered good. Moreover, serum 25(OH) D was not different in between these two groups, which supports the idea that exposure with a very low UVB dose to a very small body area is sufficient for significant vitamin D production [23]. Recent analysis of hourly mean UV index in major cities of Nepal (Kathmandu, Pokhara and Biratnagar) has shown the highest value of the index being recorded at noon-hour time for all seasons [24]. Therefore, an already established recommendation of sensible sun exposure (5–10 min of exposure of the arms and legs or hands, arms and face, 2–3 times/week) [5] between the hours of 11 00 to 14 00 has to be advocated in Nepalese population round the year to achieve vitamin D sufficiency (that maximizes endogenous vitamin D production with least possible skin damage), and it has been supported by a study done at comparable latitude in India, the neighboring country of Nepal [25]. However, increased dietary and supplemental vitamin D shouldn’t be overlooked, because population studies have consistently demonstrated high prevalence of hypovitaminosis D in Indian subcontinent despite abundant sunshine [1].

Renal transplant corrects the states of 1-α hydroxylation and hyperparathyroidism over a period of 6 months to one year, and hence the categorization of the RTRs into recently transplanted (≤ 1 year) and long term transplant recipients (> 1 year) in this study. There was no significant difference in the median serum 25(OH) D between the groups. Our study also didn’t suggest a significant relationship between serum 25(OH) D and eGFR, the finding implying that improvement of GFR in RTRs was not associated with improvement in the 25(OH) D statuses, which was in accordance to the study by Farmer et al. [26].

This study, interestingly, shows that 57.5% of the transplant population had hypocalcemia and there were no subjects with hypercalcemia. Studies have shown a variable prevalence (11–66%) of hypercalcemia following renal transplantation depending on the time post-transplantation [27]. The possible explanations could be a) a shorter pre-transplant dialysis period and b) strict dietary restrictions for RTRs (consumption of food low in calcium). This might have important clinical implication in the Nepalese RTRs. According to the recent guidelines, the use of calcitriol and vitamin D analogues is reserved in patients with CKD G4-G5 and also RTRs because of the associated increased risk of hypercalcemia and cholecalciferol supplementation has been suggested as an alternative with a lower risk. As the majority of our RTRs had hypocalcemia, it might be appropriate to supplement them with vitamin D to extract maximum benefits, without increasing the risk of hypercalcemia.

Hypovitaminosis D and its correction might be very relevant to RTRs for several reasons. The deficiency status may causeclinical symptoms such as myopathy, fatigue, muscle and bone pain. The recipients may be exposed to higher risk of bone resorption and fractures, which are further complicated by glucocorticoid-induced osteoporosis [28]. A follow up study evaluating the long term implications of vitamin D deficiency in 435 stable RTRs showed low 25(OH) D levels independently associated with an increased risk of all-cause mortality and severe deficiency, in particular, associated with a rapid annual eGFR decline [29]. Preclinical researches have shown promising outcome of VDR agonists, promoting innate immunity (thereby improving the ability of the host to combat invading pathogens) and preventing chronic allograft rejection by facilitating tolerance induction, which so far, remains an important unmet problem in RTRs [30]. Recently, vitamin D supplementation has been suggested in the treatment of kidney transplant bone disease [12] and also in post-transplant fatigue, that might improve their quality of life [31].

Hypovitaminosis D tends to be overlooked, in both CKD patients and RTRs, who are treated only with alphacalcidiol by a lot of physicians. It has to be borne in mind that adequate serum 1,25(OH)_2_D_3_ is not a substitute for inadequate serum 25(OH) D as it has negative effect on the extra-renal, locally regulated synthesis of 1,25(OH)_2_D_3_ [17]. Effective dietary vitamin D sources are very scarce and therefore, prudent exposure to sunlight and vitamin D supplementation (taking into consideration the difference in biological potency between available vitamin D_2_ and D_3_ supplements) seems to be the only feasible means to improve and correct vitamin D deficiency status in Nepalese RTRs. The optimization of supplementation however should be guided by serum 25(OH) D and other calcemic parameters because vitamin D excess may lead to hypercalcemia, hyperphosphatemia and hypercalciuria, all of which has inverse relationship with graft function [15]. A recent study in UK has concluded vitamin D repletion (using a 6 month bolus intermediate dose schedule) to be safe and effective in stable RTRs, however the post-repletion fall in vitamin D status in the absence of maintenance supplementation was intriguing [32]. Further interventional studies are warranted to explore the implications of low vitamin D status in Nepalese RTR population and whether supplementation is really beneficial and is able to sustain the vitamin D status when coupled with sensible sun exposure.

The major limitation of this study was its cross sectional design and the smaller sample size, that resulted due to limited funding available for the biochemical measurements, especially for 25(OH) D and iPTH measurements. Interesting associations between 25(OH) D and other calcemic parameters have been observed, but a prospective, longitudinal study is required to confirm if such relationship truly exists over a period of time. We also didn’t take a proper dietary history that could reveal the average intake of vitamin D in the population. This study fails to describe the seasonal variation of serum 25(OH) D that has been apparent in large population studies worldwide because the study was conducted over a short period of time (that included winter and spring seasons) the. Because of the low power of the study resulting from a small sample size, the findings in this study particularly the absence of hypercalcemia in the RTRs and absence of the significant effects of gender, BMI, sunlight avoidance behavior and post-transplant duration on the vitamin D status might not be generalizable to the large population of RTRs, and it warrants for a bigger size study.

## Conclusions

The current study reports the high prevalence of vitamin D insufficiency in South Asian RTRs, irrespective of the time after transplantation, eGFR and sunlight avoidance behavior. In particular, the negative association between serum 25(OH) D and iPTH and absence of hypercalcemia in this group could suggest the safe implementation of vitamin D supplementation for ameliorating secondary hyperparathyroidism, along with advocacy for proper and sensible sun exposure for optimization of endogenous vitamin D synthesis.

## Abbreviations

25(OH) D: 25-hydroxyvitamin D
Alp: Alkaline phosphatase
BMI: Body Mass Index
CKD: Chronic Kidney Disease
eGFR: Estimated Glomerular Filtration Rate
FGF-23: Fibroblast Growth Factor-23
iPTH: Intact parathyroid hormone
NKF/KDOQI: National Kidney Foundation Kidney Disease Outcomes Quality Initiative
NS: Statistically non-significant
RTR: Renal Transplant Recipient
VDR: Vitamin D receptor
